# Extended amygdala connectivity changes during sustained shock anticipation

**DOI:** 10.1038/s41398-017-0074-6

**Published:** 2018-01-31

**Authors:** Salvatore Torrisi, Adam X. Gorka, Javier Gonzalez-Castillo, Katherine O’Connell, Nicholas Balderston, Christian Grillon, Monique Ernst

**Affiliations:** 1 Section on the Neurobiology of Fear and Anxiety, National Institute of Mental Health, Bethesda, MD USA; 2 Section on Functional Imaging Methods, Laboratory of Brain and Cognition, NIMH, Bethesda, MD USA

## Abstract

The bed nucleus of the stria terminalis (BNST) and central amygdala (CeA) of the extended amygdala are small, anatomically interconnected brain regions. They are thought to mediate responses to sustained, unpredictable threat stimuli and phasic, predictable threat stimuli, respectively. They perform these operations largely through their interconnected networks. In two previous studies, we mapped and contrasted the resting functional connectivity networks of the BNST and CeA at 7 Tesla with high resolution. This follow-up study investigates the changes in functional connectivity of these structures during sustained anticipation of electric shock. Results show that the BNST and CeA become less strongly coupled with the ventromedial prefrontal cortex (vmPFC), cingulate, and nucleus accumbens in shock threat relative to a safety condition. In addition, the CeA becomes more strongly coupled with the thalamus under threat. An exploratory, whole-brain connectivity analysis reveals that, although the BNST/CeA exhibits generally decreased connectivity, many other cortical regions demonstrate greater coupling under threat than safety. Understanding the differential network structures of these two regions and how they contribute to processing under threat will help elucidate the building blocks of the anxious state.

## Introduction

Defensive responses are fundamental to survival, and extensive basic and clinical research has been devoted to study the neural mechanisms underlying their expression and regulation^1–3^. However, current understanding remains elusive, because of the complexity of the multiple processes involved and the difficulty of capturing small structures with standard neuroimaging tools. The emergence of more sophisticated research tools such as ultra-high field (UHF, 7 Tesla) magnetic resonance imaging can palliate the latter issue.

A number of heuristic models have helped guide research on defensive behavior. One such model, the dual model of defensive responses, recognizes two main types of responses: a quick automatic response to a proximal threat and a sustained response to a distal or uncertain threat^4^. The former quick response, fear, depends on a distributed circuit centered on the central nucleus of the amygdala (CeA)^5^, whereas the latter sustained response, anxiety, is associated with an overlapping circuit that depends on the bed nucleus of the stria terminalis (BNST)^4^. This dichotomy, which contrasts amygdala/fear to BNST/anxiety, still needs validation^6–8^, and the lag in knowledge partly reflects the limitation of conventional magnetic resonance imaging (MRI) to reliably investigate small structures in humans. A better delineation of how these structures communicate with the rest of the brain may address the functional commonalities as well as uniqueness of these structures in the encoding of fear and anxiety processes. The present study takes advantage of UHF-functional MRI (fMRI) to assay the responses to sustained threat of the functional circuitries of the BNST and the CeA.

Specifically, this study focuses on how an anxious state alters the intrinsic functional connectivity (IFC) of the BNST and CeA across the brain. IFC refers to the coupling of spontaneous oscillations of BOLD activity among brain regions^9^. Interest in this measure has exploded because of the ease of acquisition, short duration (6–12 min), and the remarkable analogy of the canonical resting networks with sets of co-activated regions associated with specific task domains^10^. In addition to being influenced by age (development and aging), sex, or pathology, this functional architecture is also sensitive to transient-state modifications, such as task-related cognitive processes or emotional states such as anxiety^11,12^.

Reports detailing the anatomical connectivity of the extended amygdala originate from basic research in rodents and non-human primates that assign the CeA and BNST to the position of stations that integrate information from sensory domains and dispatch it back to distributed networks. The BNST and CeA are reciprocally interconnected^13^ and share afferents from other amygdala subnuclei (e.g., basolateral complex), as well as efferents to targets such as hypothalamus and midbrain (e.g., substantia nigra and periaqueductal gray)^14,15^. The CeA and BNST are also connected with other systems, i.e., subcortical (striatum, thalamus, and hippocampus) and cortical (primary sensory and associative cortices, insula).

Although a number of human neuroimaging studies have examined the connectivity and function of the extended amygdala using conventional 3 T MRI in task as well as no-task designs^16–20^, the present work is most closely informed by findings from two recent resting-state fMRI studies that describe the IFC of BNST and CeA using UHF fMRI^21,22^. Building on Torrisi et al.^21^ and Gorka et al.^22^ demonstrated that BNST and CeA IFCs overlapped, covering cortical (i.e., ventromedial and dorsomedial prefrontal cortex (PFC)) and subcortical (i.e., centromedial thalamus, periaqueductal gray, hippocampus, and nucleus accumbens (NaC)) regions. However, a direct contrast of CeA and BNST IFCs identified distinct targets. Specifically, the BNST showed stronger connectivity to regions involved in cognitive and motor processes, i.e., areas of cingulate cortex and caudate nucleus, whereas the CeA showed stronger connectivity to regions involved in sensory and attention processing, i.e., insula, posterolateral thalamus, and fusiform gyrus. These findings may fit with the putatively distinct role of the BNST in sustained defensive responses, which preferentially engage cognitive processes (e.g., worry) and goal-directed motor responses, and the CeA in emergency defensive responses, which presuppose rapid integration of multiple sensory inputs to drive automatic behavior. Such interpretation is limited by the neutral, resting context of the data, when anxiety and sustained defensive response networks were not actively engaged. The present study tests the hypothesis that the regional connectivity most affected by sustained threat would be a subset of these CeA and BNST connections. The BNST IFC is expected to be more affected by sustained threat, relative to CeA IFC, particularly those targets that are more strongly coupled with the BNST (i.e., striatum and cingulate cortex).

The direction of changes (higher or lower measures of coupling) in IFC is difficult to predict because of the unique design of the present work. Most human studies reporting on defensive behaviors use event-related task fMRI^23^. Here, coupling among spontaneous BOLD oscillations is the measure of interest. Notably, the direction of changes in event-related BOLD activations has a complicated and as yet not fully understood relation to changes in connectivity^24^. The closest previous work to the present study is from Vytal et al.^25^, who used a similar design with a 3 T scanner, and examined the IFC of the entire amygdala during sustained periods of threat and safety. Findings of threat vs. safety contrast revealed decreased amygdala connectivity with the ventromedial PFC and precuneus, and increased amygdala connectivity with the thalamus and insula.

On the basis of the differential connectivity and putative functions of the CeA and BNST and previous work, hypotheses are two-fold. First, we predict that BNST connectivity with upstream structures, serving higher-order functions (i.e., prefrontal regions), would be preferentially affected in sustained threat, which engages cognitive resources. Second, based on Vytal et al.^25^ we predict patterns of both strengthening and weakening of regional connectivity, specifically strengthened CeA–thalamus and CeA–insula couplings and weakened CeA–vmPFC and CeA–precuneus couplings.

## Materials and methods

### Subjects

Forty-three right-handed, healthy volunteers from a mixed urban and suburban population were recruited and were compensated for their time. Exclusion criteria included the following: (a) current or past Axis I psychiatric disorder as assessed by the Structured Clinical Interview for DSM-IV, Non-patient Edition (SCID-I/NP)^26^, (b) first-degree relative with a known psychotic disorder, (c) brain abnormality on MRI as assessed by a radiologist, (d) positive toxicology screen, (e) MRI contraindications, or (f) excessive head motion during scanning. Excessive head motion, defined below, led to removing seven subjects, leading to a final *N* = 36 (15 female subjects, mean (SD) age = 28.4 (6.0) years). Race demographics were as follows: asian: 22%, black: 19%, mixed: 8%, white: 42%, and others: 8%. The mean (SD) Hollingshead social economic status is 59.9^11^. The mean WASI IQ is 118.3^12^; however, we note that six subjects were missing WASI data. The subjects were screened for current drug use and were asked to abstain from drinking caffeinated beverages and smoking for at least an hour prior to testing. None of the subjects smoked tobacco or used illegal drugs, and those who drank alcohol consumed less than seven drinks per week. In addition, the subjects were instructed not to drink alcohol on the night prior to and the day of testing. Subjects gave written informed consent, which was approved by the National Institute of Mental Health Combined Neuroscience Institutional Review Board.

### Functional image acquisition

Scanning was performed on a 7 T Siemens Magnetom MRI with a 32-channel head coil. The high-resolution, 0.7 mm isotropic MPRAGE was T1-weighted with the following parameters: TR = 2200 ms, TE = 3.01 ms, and acquisition matrix = 320 × 320. Flip angle was 7°. The functional echo planar images (EPI) had a repetition time (TR) = 2.0 s, echo time (TE) = 27 ms, flip angle 70°, 1.4 mm isotropic voxels, and 45 slices, with 490 images collected over 16 min and 20 s. Non-whole-brain field of view (FOV) was angled to capture amygdalae and the dorsal anterior cingulate cortex.

### Threat of shock condition

Subjects were instructed to keep their eyes open and look at a black fixation cross on a white background with one or two colored borders. A blue border indicated that they were safe from shock and a red border indicated that they were under threat of shock. The design of the task consisted of 10 s of no border at the start and end of the scan, and 16 min of colored borders divided into four, 4-min blocks of alternating red or blue. There was no break between colored borders, and the color of the first border was counterbalanced across subjects. To probe the “sustained” nature of anxiety, 4-min blocks were chosen as a compromise between a length of time estimated to promote minimal habituation and fading of threat anticipation, and one that provided enough within-condition data points for stable connectivity calculations.

Because there were no breaks between condition blocks, the inner edge time points of the blocks were subsequently removed from the analysis. These consisted of six TRs on each end (12 TRs or 24 s removed within each block, i.e., 96 s total removed from the 16 min scan). Within-block time point removal resulted in correlations across 384 s of the safety or threat conditions (6.4 min each). This was to remove psychological and BOLD bleed-over between conditions, as well as to minimize the effects of shock delivery, which were near the start or end of blocks.

### Shock procedure

The threat of shock protocol was based on a translational psychophysiology paradigm^27^ adapted for neuroimaging (e.g. ref. ^25^). The NIH MRI Safety Board determined that shock to the wrist in the 7 T environment was safe because the transmit coil was head-only. Prior to the task, subjects completed a work-up procedure to control for individual differences in shock tolerance and to titrate the intensity to a level that was highly uncomfortable and aversive, but tolerable. Subjective ratings ranged from 1 (barely felt) to 5 (painful), with a mean of 4.5. To ensure shock unpredictability, subjects were told that, during the red-bordered blocks, the computer would randomly deliver between one and five shocks. In actuality, a single 100-ms shock was delivered to the left wrist (DS7A; Digitimer, UK) in each of the two threat blocks (i.e., two shocks total) at pre-determined times within 10 s of the beginning or end of a threat block. Efficacy of threat manipulation was verified via a post-session retrospective rating of “how anxious” they felt during the safety and threat conditions (1 (not at all)–10 (extremely) Likert scale).

### Physiological measures

For physiological noise removal, respiration was measured with a belt around the diaphragm, and cardiac pulse was measured with a pulse oximeter on the right index finger. Physiological data were sampled at 500 hz using AcqKnowledge software connected via a BioPac MP150 system (www.biopac.com). An error prevented recording the respiration of one subject, for whom only cardiac regressors were used.

### Mask definitions

For the BNST, three authors (S.T., K.O., and A.D.) separately drew BNSTs in AFNI on subjects’ native-space structural images, as described in ref. ^21^. Consensus masks for each individual, representing a thresholded average of the three authors, were used for time-series extraction.

The CeA mask was derived from a probabilistic mask created from multimodal (T1-weighted and T2-weighted) high-resolution structural scans^28^. We thresholded these at 20%, checked the center of mass of each subnucleus against an atlas in MNI space^29^, and examined their alignment with an MNI template^30^ as well as an average of each individual’s normalizations to that template.

### Preprocessing and main analyses

Preprocessing was similar to our resting-state analysis^21^. Tissue segmentation was performed with FreeSurfer^31^. Functional data preprocessing and analyses were performed within AFNI^32^ using the ANATICOR processing stream^33^, which circumvents problems resulting from regressing out a global signal^34,35^. In brief, subjects’ first four functional volumes were removed and the remaining functional volumes were slice-time and motion-corrected and co-registered to their MPRAGE structurals. Subjects’ anatomy was then nonlinearly normalized to the template using *3dQwarp*^36^, with the resulting parameters applied to the hand-drawn BNSTs, segmentations, and the functional data. Functional data were smoothed with a 2.8 mm FWHM kernel.

Noise regressors were modeled as covariates of no interest in a single regression and were projected out of the data to leave cleaned time series maps. These regressors of no interest included head motion parameters and their first derivatives, shock events, their time and dispersion derivatives^37^, 13 slice-based cardiac and respiration phase regressors (RETROICOR) and respiration volume per unit time measures^38,39^, and lateral and 3^rd^ + 4^th^ ventricle masks. Excessive head motion, resulting in the removal of seven subjects, was defined as more than 10% of a subject’s images censored, where the criteria were a motion derivative greater than 0.3 mm for temporally adjacent time points (1.3 ± 2% of TRs for remaining 36 subjects). The data were high-pass-filtered (up through third-order polynomials) to remove low-frequency drift up to, but not including, the frequency of the four-condition blocks. Incidentally, high-pass-filtering, rather than bandpass-filtering, has been shown to improve signal noise separation, test–retest reliability, and to avoid artificial autocorrelations^40,41^. Noise was conservatively removed from white matter using the ANATICOR approach. This was performed separately for every gray matter voxel by regressing out the averaged time series of white matter found within a 14 mm radius sphere surrounding a given gray matter voxel. This allowed for the removal of variance from white matter spatial heterogeneity, as well as potential hardware-related artifacts^33^. Finally, to examine the issue of signal dropout at 7 T, the air/tissue interfaces were visually compared at the group level to a 3 T data set with standard acquisition parameters^42^. We re-normalized the 3 T data set with the same nonlinear algorithm and to the same template employed here. Results support previous work demonstrating that acquiring thinner slices mitigates signal dropout^43,44^ and is less at 7 T (Supplementary Fig. 1).

Using averaged time series extracted from the bilateral BNST and CeA masks, correlations across the brain were computed and Fisher-transformed. Two-tailed, one-sample *t*-tests were separately performed for the BNST and CeA safety condition maps to verify consistency with previously reported resting connectivity of these seeds^21,22^. Two two-tailed, paired *t*-tests were then separately performed for CeA and BNST to assess differences in resting connectivity between threat and safety conditions. Voxel-level multiple comparison correction was set at *p* < 0.005, *k* = 54 (cluster-level corrected at *p* < 0.05), determined with *3dClustSim* using group-averaged smoothing estimates calculated with *3dFWHMx*. The gray matter mask used for multiple comparison correction and for masking group-level results was a 25% probability gray matter mask from the template that had been cropped to a field-of-view representing 95% or more of subjects’ EPI coverage (see Supplemental Fig. 2).

#### Exploratory analysis: functional correlation matrix

To place into perspective and complement the a priori seed analyses of the BNST and CeA, an exploratory multicorrelation analysis was conducted on 109 regions of interest (ROIs) across the brain. Briefly, the analysis used FreeSurfer, AFNI^45,46^, and custom scripts to calculate a mean correlation score for each ROI with all the other ROIs in each specific condition, threat, or safety. The ROIs were selected by first combining the dense Destrieux et al. cortical parcellation with a number of a priori subcortical ROIs, and then systematically pruning this large number of ROIs to those that were sufficiently contained within the group-level mask and had good temporal signal to noise. Further detail can be found in the supplemental materials. Threat effects (threat minus safety) were tested using paired *t*-tests in MATLAB (MathWorks Inc., Natick, MA), which were conducted on the condition-specific Fisher-transformed correlations.

Because of the large number (109×108)/2 = 5886 of tests on network edges, the threat vs. safety comparison of the correlation matrices did not yield statistically significant results at the conventional *p* < 0.05 threshold after Bonferroni or FDR corrections for multiple comparisons. Therefore, a “mean” approach was taken on each region’s connectivity. This was performed by calculating the mean of the weighted correlations of each region, i.e., the mean across matrix columns, to obtain a metric of overall connectedness difference (threat>no threat) for each of the 109 ROIs. This approach calculates what is also called a node’s “strength”^47^. In other words, a positive mean metric for a given region represented, on average, higher coupling during the threat than safety across all other 108 ROIs. Likewise, a negative mean metric for a given region represented, on average, higher coupling during safety across all other 108 ROIs. An arbitrary threshold was selected at >1 and <1 standard deviation away from the mean of the 109 summation metrics to identify ROIs with the strongest connections under threat and the strongest connections under safety. Although this approach essentially ignores regions with mixed connectivity profiles (where they would average closer to zero), it provides a broad picture of threat effects on global functional organization.

## Results

### Threat manipulation

Retrospective self-reports confirmed the efficacy of the threat manipulation. The 1–10 Likert scale indicated significantly greater retrospective anxiety during the threat compared to the safety condition (pre = 2.09 (1.1), post = 6.2 (2.0); *p* < 0.0001).

### Within-seed BNST and CeA functional connectivity during safety

**Fig. 1:**
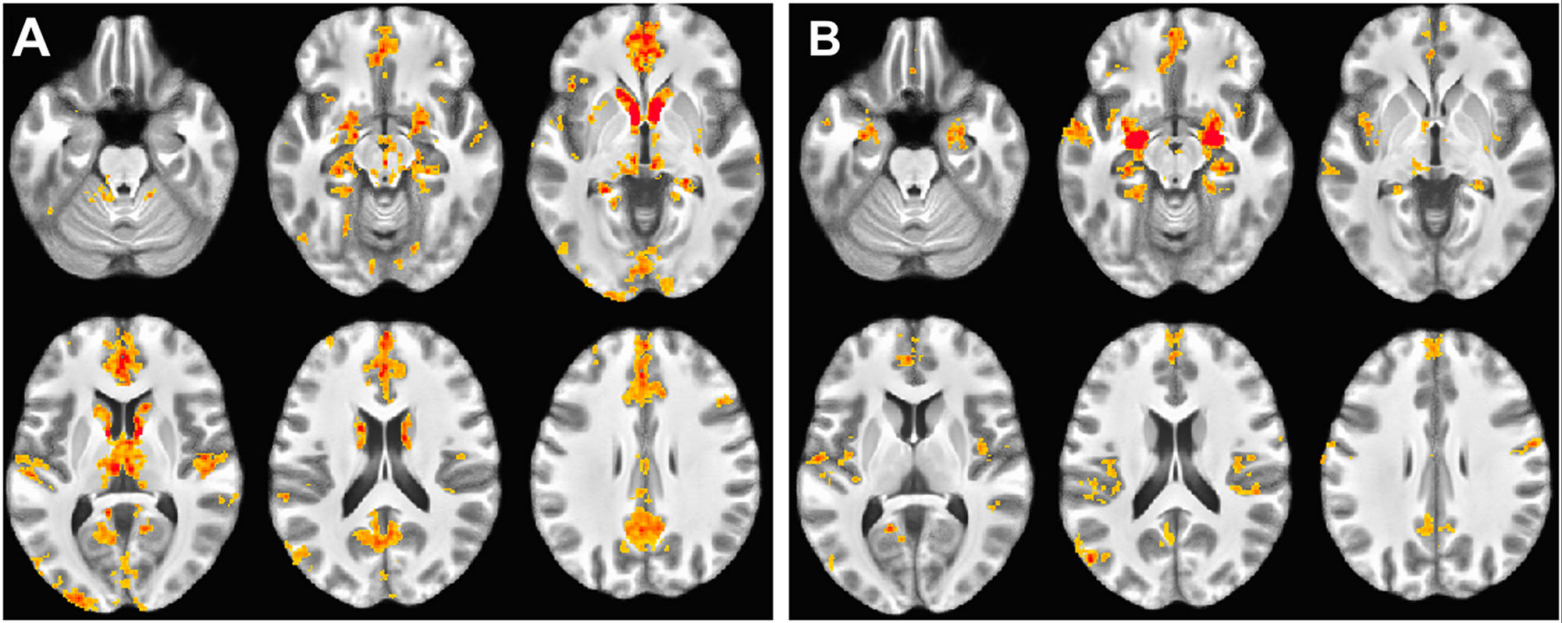
Resting connectivity during safety blocks. **a** BNST functional connectivity. **b** CeA functional connectivity. Results thresholded at *p* < 1 × 10^−7^, *k* = 100. Results in these and subsequent figures are overlaid on the average of subjects’ structural scans

The safety condition was used to confirm replication of previously published resting-state results. Figure 1a (left) demonstrates significant positive BNST coupling with regions previously reported in ref. ^21^, including most prominently the dorsal amygdala, hippocampus, periaqueductal gray, thalamus, head of the caudate, anterior insula, ventromedial PFC (vmPFC), medial PFC (mPFC), and precuneus.

Figure 1b demonstrates significant positive CeA coupling with regions previously reported in ref. ^22^, including the BNST, vmPFC, mPFC, fusiform gyrus, and mid- and posterior insula.

### BNST functional connectivity, threat vs. safety

**Fig. 2:**
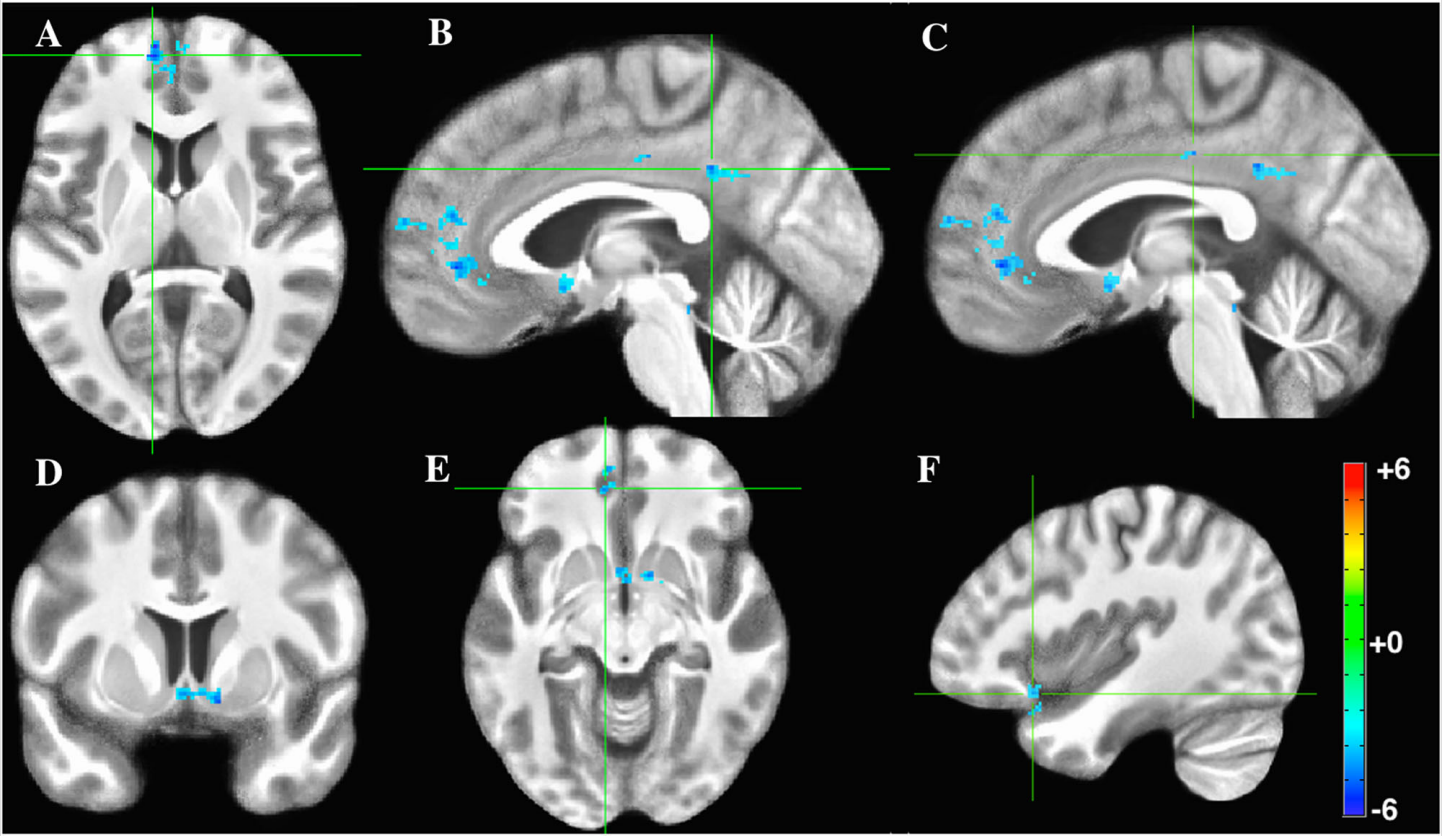
BNST connectivity, threat vs. safety contrast. **a** Axial slice of medial prefrontal cortex (mPFC). **b** Sagittal slice of posterior cingulate. **c** Sagittal slice of posterior cingulate. **d** Coronal slice of nucleus accumbens. **e** Axial slice of ventromedial PFC (vmPFC). **f** Sagittal slice of ventrolateral prefrontal cortex (vlPFC). Figure thresholded at *p* < 0.005, *k* = 54. Note that here negative *t*-statistics represent greater positive connectivity during the safety condition, and not negative connectivity

Figure 2 displays the six regions that demonstrate significantly less positive coupling with the BNST during threat than safety. These regions included the mPFC, precuneus, NAc, and left vmPFC (Table 1). No regions showed significantly greater connectivity with the BNST during threat than safety.

**Table 1:** Paired *t-*tests between conditions (threat > safety), with BNST and CeA correlation maps separately reported

Region	*X*	*Y*	*Z*	Cluster size	*t*-stat
*BNST: threat vs. safety*					
Left superior medial frontal gyrus (BA 10)*	−10.3	57.1	8.2	801	−5.79
Left posterior dorsal cingulate gyrus (BA 23)	−3.3	−43.7	33.4	160	−4.65
Right nucleus accumbens	12.1	6.8	−10	114	−4.96
Left ventrolateral PFC (BA 47)	−42.5	19.3	−15.6	59	−4.29
Left ventromedial PFC (BA 11)	−8.9	44.6	−8.6	55	−4.29
Left posterior cingulate gyrus	−3.3	−21.2	37.6	55	−4.28
*CeA: threat vs. safety*					
Left ventromedial PFC (BA 11)	−8.9	45.9	−7.2	64	−4.99
Right ventroanterior thalamus	12.1	−7.2	9.6	61	5.37

Coordinates in MNI space. *BA* (approx.) Brodmann Area. *indicates additionally whole brain corrected at *p* < 0.05 using *p* < 0.001 cluster-defining threshold

### CeA functional connectivity, threat vs. safety

**Fig. 3:**
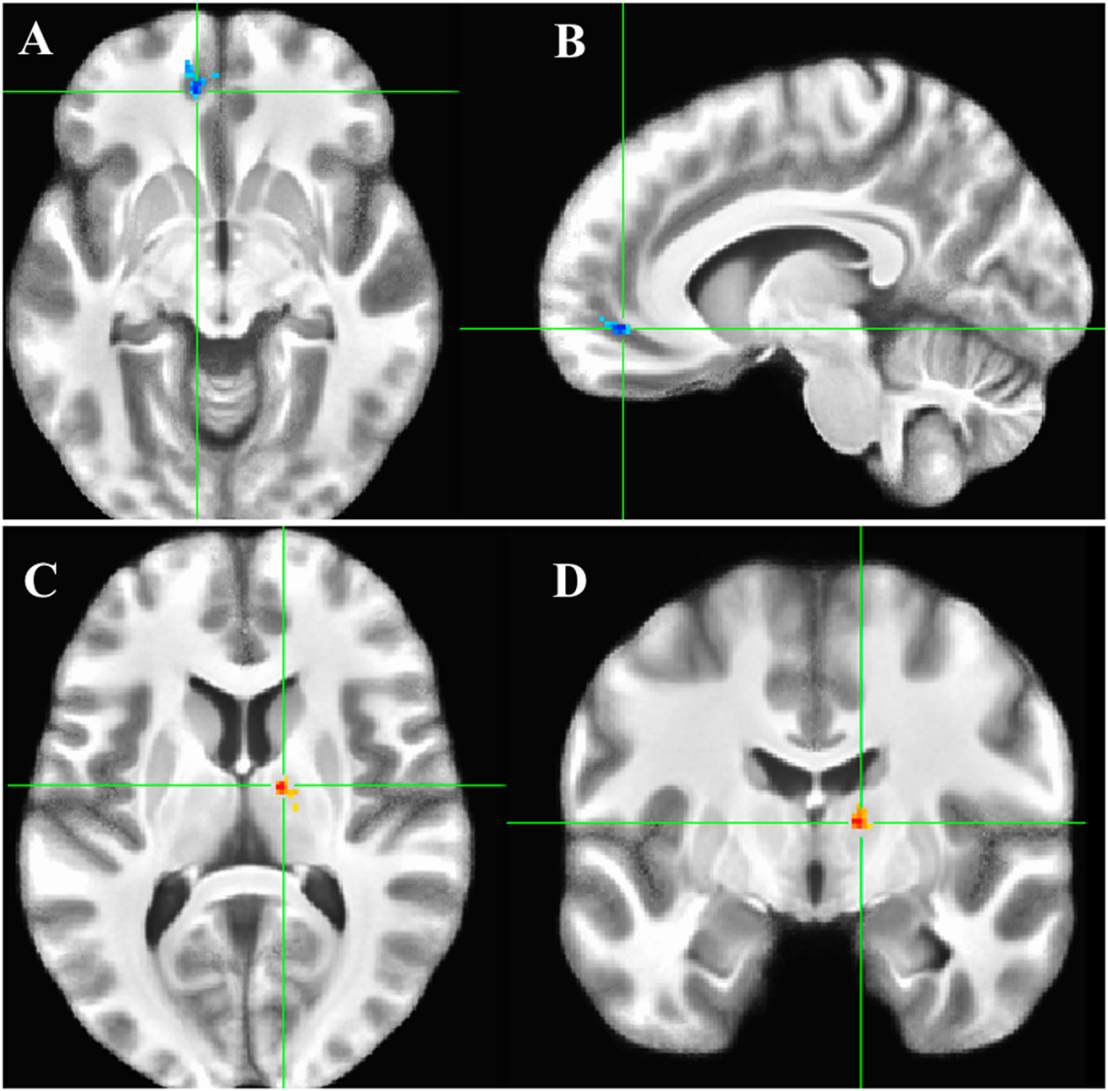
CeA connectivity, threat vs. safety contrast. Axial slice results: **a** ventromedial prefrontal cortex (vmPFC). **b** Right ventral–anterior nucleus of the thalamus. Color scheme is the same as Fig. 2

Figure 3 displays two regions within the vmPFC and thalamus that demonstrate a significant difference in functional coupling with the CeA between conditions (Table 1). The left vmPFC cluster (Fig. 3a) is less positively coupled with the CeA during threat than safety. In contrast, a ventral–anterior thalamic cluster, located lateral to the internal medullary lamina^29^, is more strongly coupled with the CeA during threat than safety.

### Exploratory matrix analysis

**Fig. 4:**
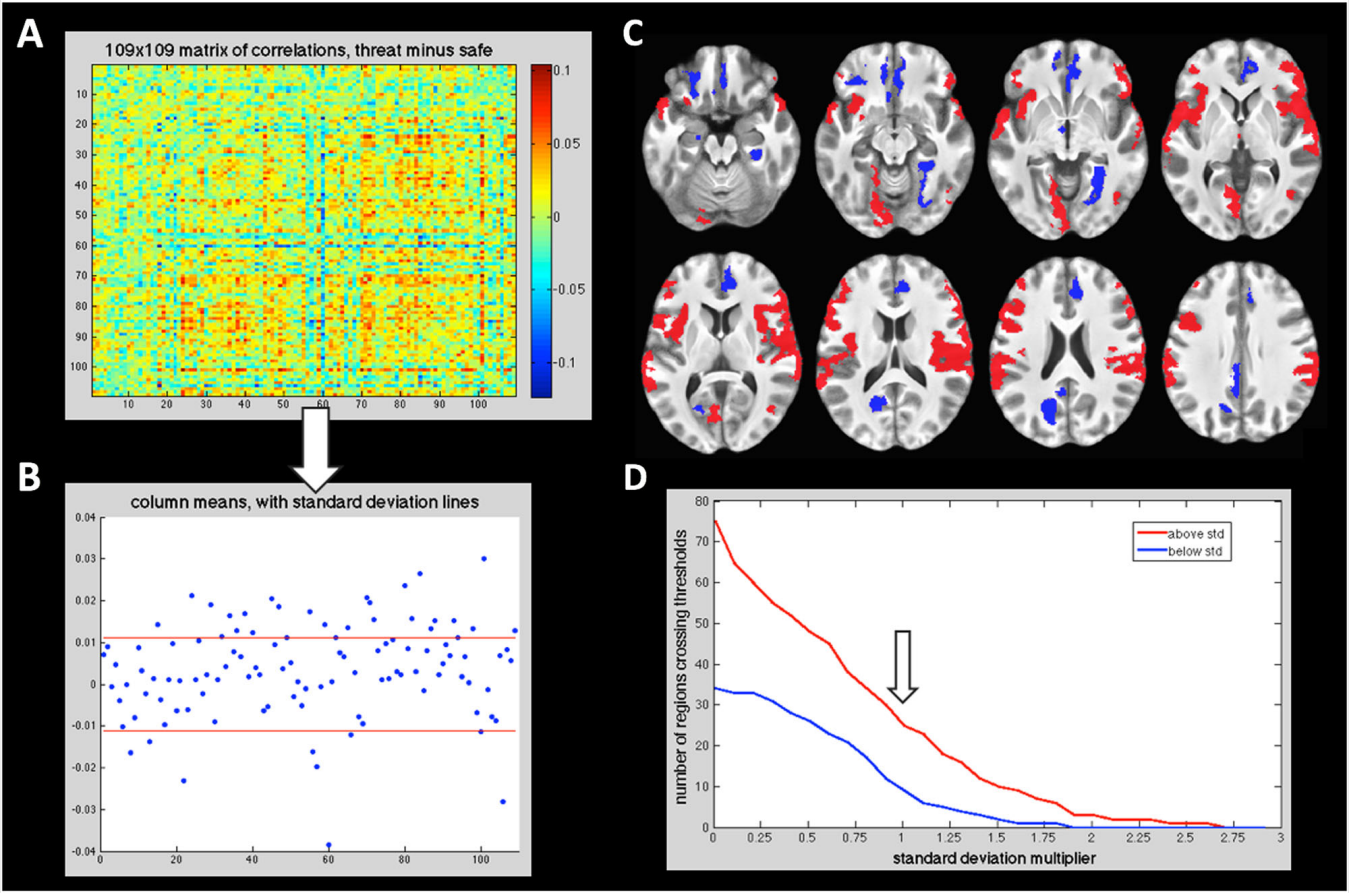
Qualitative exploratory analysis. **a** 109 region correlation matrix, as contrasted Threat > Safety. **b** Mean correlations (i.e., 4 A matrix columns collapsed) with SD lines in red. See Table 2 for regions above and below 1 SD from mean. **c** Ventral to dorsal axial slices of > ± 1 SD regions visualized on the brain: positive correlation regions red, negative correlation regions blue. **d** Thresholding in (**b**) and (**c**) was arbitrarily set at 1 SD; however, the observation that *more* regions were on average more positively connected during threat than safety is invariant to thresholding (red greater than blue line across thresholds: down arrow points to 1 SD point)

Figure 4a represents the functional connectivity matrix of the whole brain (within FOV) as a paired *t*-test of threat vs. safety. A threshold of >1 and <1 standard deviation from the mean of 109 ROI mean correlations was then selected to identify the most robust effects of threat for each region (Fig. 4b). Negative difference scores (from threat minus safety) were the strongest in bilateral vmPFC and precuneus, and positive difference scores (also from threat minus safety) were the strongest in vlPFC, IFG, anterior insula, centromedial thalamus, and other temporal–parietal regions (see Table 2 for the full list of regions at this threshold). Table 2 lists the regions with the highest absolute mean IFC during threat (*n* = 25) and during safety (*n* = 9). Figure 4c shows a montage of axial slices visualizing these ROIs. We observe that a greater number of regions crossed the positive threshold rather than the negative threshold. While the absolute number of regions is threshold-dependent, Fig. 4d further demonstrates that this basic observation, of a greater number of more-positively connected regions during threat, is threshold invariant.

**Table 2:** Regions from exploratory matrix “mean correlation” analysis

Positive connectivity regions	BA	X	Y	Z	*µ* corrs
Right orbital lateraleral sulcus	47	45	43	−6	0.0301
Right temporal superior planar gyrus	42	60	−28	18	0.0266
Right parietal inferior supramarginal gyrus	40	63	−27	28	0.0235
Left front inferior opercular gyrus	44	−53	12	9	0.0211
Right front inferior opercular gyrus	44	54	13	5	0.0207
Left anterior insula circular sulcus	13	−31	21	−11	0.0205
Right front inferior orbital gyrus	47	50	35	−13	0.0196
Left insular short gyrus	13	−40	10	−5	0.0189
Left superior circular insula sulcus	13	−33	28	2	0.0186
Left orbitolateral sulcus	11	−46	42	−8	0.0172
Left temporal superior Plan temporal gyrus	42	−60	−35	16	0.0168
Left pariet inferior supramarginal gyrus	40	−61	−30	24	0.0163
Right temporal superior lateral gyrus	21	60	−4	−5	0.0158
Right frontoinferior triangul gyrus	45	55	32	4	0.0155
Right circular insula superior sulcus	13	37	7	9	0.0152
Right lateral fissure posterior	13	40	−25	18	0.0151
Left precentral−inferior sulcus	44	−50	5	24	0.0144
Centromedial pf of thalamus		−1	−13	2	0.0143
Subcentral gyrus and sulcus	43	58	−7	14	0.0135
Right occipital anterior sulcus	37	45	−65	3	0.0133
Right lateral fissure anterior vertical	45	48	22	9	0.0132
Left temporal superior lateraleral gyrus	21	−61	−9	−3	0.0129
Right temporal transverse sulcus	22	53	−18	7	0.0129
Left lateral fissure anterior horizontal	47	−42	31	−3	0.0124
Left occipitotemporal medial lingual gyrus	18	−17	−69	−12	0.0114
Left inferior frontal sulcus	46	−39	39	15	0.0112
*Negative connectivity regions*	BA	X	Y	Z	µ corrs
Right sulcus oc-temporal med and lingual	37	29	−44	−12	−0.0115
Right anterior cingulate gyrus and sulcus	32	4	43	2	−0.012
Hypothalamus		−5	−7	−6	−0.0138
Left orbital H-shaped sulcus	11	−28	39	−17	−0.0161
Left anterior hippocampus		−20	−14	−19	−0.0165
Left parieto-occipital sulcus	31	−16	−66	21	−0.0197
Left posterior–dorsal cingulate gyrus	31	−4	−42	29	−0.0232
Right suborbital sulcus	11	4	45	−18	−0.0281
Left suborbital sulcus	11	−7	32	−15	−0.0384

These are regions that survived the >1 or <1 standard deviation thresholds (Figs. 4b, c). Note, however, the threshold dependence of such an approach (Fig. 4d). Region labeling primarily from ref. ^45^ with the exception of Brodmann’s Areas. Center of mass coordinates in MNI space, based on thresholded, group-averaged regions. Regions are sorted by strength of mean correlations. *BA*(approximate) Brodmann’s area

## Discussion

A recent debate questions the distinct role of the CeA and BNST in the two main types of defensive responses, fear and anxiety^6–8^. One way to address it is to examine responses of the intrinsic functional networks of the CeA and BNST to anxiety manipulation. To this goal, the present study uses the high spatial resolution that UHF affords to focus on CeA and BNST responses to sustained threat (anxiety). Predictions were two-fold. First, threat-related alterations in BNST couplings were expected to involve regions supporting cognitive function, such as the prefrontal and cingulate cortices, by virtue of cognitive engagement in anxiety^48^. Second, based on amygdala findings in a similar study^25^, the CeA–thalamus and CeA–insula couplings were anticipated to be stronger under threat than safety, and CeA–vmPFC and CeA–precuneus to be weaker. As discussed below, findings were broadly in line with these hypotheses.

### Direction of BNST and CeA IFC changes

In the main analysis, the direction of BNST-IFC changes to sustained threat was a uniform decrease of connectivity strength for all statistically significant clusters. Such IFC reduction was in contrast to the observation that, from the whole-brain exploratory summation metric, regions with strongly increased IFC (*N* = 25) were more numerous than those with strongly decreased IFC (*N* = 9; Figs. 4b, c). Although the absolute number of regions showing a summation metric of strongly increased or decreased connectivity was dependent on thresholding, an additional post hoc test demonstrated that the basic finding of a greater number of positively coupled than negatively coupled regions was invariant to threshold (Fig. 4d). Although the meaning of weaker IFC connectivity at the physiological levels is not yet fully understood, a number of mechanisms can be suggested.

First, the standard Pearson correlations between regions calculated herein reflect “static” functional connectivity across the entire length of the condition blocks (~4 min, averaged between two blocks of each condition). Any systematic fluctuations in connectivity that occur at shorter timescales are consequently not reflected. Such shorter fluctuations (e.g., lasting 30 s rather than 4 min) may represent transient neural processes^49^, which would result in greater variability, in turn causing weaker static connectivity during a condition of interest. In fact, McMenamin and colleagues reported on temporal characteristics of functional connectivity across blocks of threat and safety conditions by using a data-driven estimation of three temporal factors^20^. Findings revealed a period corresponding with a transient period of entering threat, a second sustained period, and a late sustained period. Future work with dynamic resting-state analyses may provide further insights into threat-related reductions in connectivity strength^50^.

A second possibility explaining a threat-related reduction in IFC could be the engagement during threat processing of additional intermediary pathways not detected in the present analyses. Such regional diversification of the network would serve to increase flexibility of responses to potential threat, enhancing the recruitment of multiple regions necessary to escape harm. Within a larger network, connectivity across a primary pathway might be reduced.

Overall, weaker connectivity could reflect more flexibility and diversity within the BNST circuit, as a way to provide optimal preparedness for a potential threat. However, other interpretations, based on inferential regional functions, might be proposed, as addressed in the next section.

### Functional significance of the BNST-IFC clusters sensitive to sustained threat

The impact on BNST IFC by sustained threat revealed both cortical (PFC and PCC) and subcortical (ventral striatum) regions. In line with predictions, sustained threat affected BNST connectivity to regions supporting cognitive functions. Most prominently, both anterior (vmPFC) and posterior (PCC) medial cortical regions were modulated by threat (Table 1). The medial cortical structures may be interpreted from the perspective of the default mode network (DMN).

Although the subject of much scrutiny for well over a decade, the functioning and significance of the DMN remains elusive. On the one hand, it is detected in rodents, under anesthesia, and in fetal brains^51–53^, suggesting that it is a general organizing feature of the brain and not tied to a particular cognitive process^54,55^. On the other hand, much evidence specifically connects the DMN to social cognition and self-reflective processes^55,56^. The current setting, which can constrain functional interpretations^57^, may help uncover a functional link between the BNST and the DMN. In fact, a similar pattern of BNST-DMN connectivity has also been observed in other resting-state studies^19,21^. While not a traditional component of the DMN, the BNST may, however, have special access to it. Indeed, the threat-related reduction in BNST-DMN IFC found here could involve enhanced vigilance toward the environment. This hypothesis is supported by work consistently showing increased perceptual processing during threat of shock^58,59^. In other words, the reduction in BNST-DMN IFC would imply a shift away from endogenous attention toward monitoring the environment.

The NAc is another region whose coupling with BNST was altered by sustained threat. The NAc belongs to the limbic part of the ventral striatum, and is specifically involved in incentive-related behavior^60^. Whereas the BNST has been shown to functionally and structurally be coupled with the dorsal striatum^19,21,61^, the specific modulation of BNST-NAc with sustained threat suggests a role of the ventral striatum in response to sustained threat and anxiety as well^62,63^. In Torrisi et al., the BNST–NAc connectivity was discussed in reference to substance abuse^64^. Indeed, the hypothesis that BNST–NAc connectivity is relevant to addiction is predicated on stages of the addiction cycle that include anxiety, namely the withdrawal and negative affect stage^64^. The present data suggest that the ventral striatum could be included in the anxiety-related pathways. Although speculative, the involvement of the BNST-NAc in sustained threat might reflect the motivation to avoid, or to prepare motor responses in case the threat materializes.

### Functional significance of the CeA-IFC clusters sensitive to sustained threat

Similar to the BNST-IFC, the CeA-IFC showed a threat-related decreased coupling with the vmPFC. In fact, the CeA-vmPFC and BNST-vmPFC clusters were overlapping. This suggests that CeA and BNST have a coordinated response to sustained threat through the same vmPFC region. The role of vmPFC in emotion regulation has been well established in humans^65^. The leading theory argues for a top–down inhibition of brain regions involved in the processing of negative emotion^66^. Although a similar relationship could be hypothesized for BNST and vmPFC, recent studies have supported the opposite^67,68^. Accordingly, vmPFC drives BNST activity, which, in turn, activates the behavioral and physiological components of negative emotion^69,70^. However, this interpretation raises the question of the physiological mechanisms underlying reduced IFC, i.e., to what extent does reduced IFC reflect inhibitory and/or excitatory neural activity.

The thalamus was the other CeA-IFC cluster (positively) modulated by sustained threat. The thalamus is a complex multinucleus structure, filtering sensory information, directing attention, and modulating arousal^71^. As such, the thalamus has been implicated in the reorientation of attention to threat and the concomitant rise in arousal^72^. The strengthening of CeA–thalamus coupling might reflect the maintenance of salient information originating from the CeA, helping to keep sustained focus on potential threat. By the same token, given the reciprocal thalamus–CeA connection, this strengthened coupling might also serve to maintain the channeling of somatosensory information to the CeA. This information would pass to the BNST, which would transfer it to the NAc, and generate action responses. Previous findings indicate that both CeA and BNST are also coupled with the thalamus at rest, particularly with the midline nuclei^21,22^.

### Unexpected null results

Although null results cannot be ruled out, they are important to address for future work. Unexpectedly, neither the insula nor hippocampus, which are prominently implicated in anxiety^73,74^, exhibited altered couplings with CeA or BNST under threat. In fact, the exploratory correlation analysis of the 109 ROI matrix identified the anterior insula as one of the regions whose whole-brain IFC was among those with the most strongly increased IFC under threat (Fig. 4c and Table 2). In addition, our previous resting-state finding indicated that the insula was not part of the BNST-IFC, in contrast to its representation in CeA-IFC^21,22^. Previously, we suggested^21^ that the absence of BNST-insula IFC might reflect a lack of the anxious state during rest, and that, with an anxiety challenge, BNST-insula coupling would emerge. The present work does not support this hypothesis. Therefore, a parsimonious explanation would be that anxiety simultaneously engages independent neural circuits; one involving an insular circuit and another the BNST and CeA.

Regarding the hippocampus, this structure has often been associated with context conditioning^75^. Basic findings in rodents implicate the hippocampus in context conditioning^76^. Human studies show only *transient* but not sustained hippocampus activation during context conditioning^77–79^. Our finding that the hippocampus does not show a change in coupling with the CeA or BNST during *sustained* threat is consistent with these results.

### Strengths and limitations

Strengths include the within-subject design of the threat manipulation, the well-validated threat-induction paradigm^27^, the replication within our safety blocks, and many of our between-condition clusters of previously reported BNST and CeA resting connectivity with a different data set^21,22^, and the use of UHF imaging with a resolution that was an order of magnitude finer than standard, 3 T fMRI (2.7 vs. 27 mm^3^). However, such resolution was obtained at the expense of full-brain coverage. Full-brain coverage with finer resolution is now available using simultaneous multislice or multiband acquisitions^80^, and should be used in future studies. A second limitation concerns the voxel-level cluster-defining threshold (*p* < 0.005) that was used for multiple comparisons correction to achieve whole-brain correction at *p* < 0.05. Recent evidence suggests that this may be too liberal^81^, and that better practice would be to define clusters at *p* < 0.001. For the present work, however, we chose to favor potential false-positive rather than negative results^82^ and focus on sensitivity rather than specificity, given the novel and exploratory nature of the study. A third limitation concerns the BNST masks which could not resolve its subdivisions. However, the masks purposefully included mostly the lateral part of the BNST^21^, which is the BNST component most implicated in anxiety-related processes^4^. Finally, it was a limitation that the correlation matrix was uncorrected; however, as stated above, it is presented to contextualize the voxel-level results and to generate hypotheses for future studies.

## Conclusions

In summary, this is the first study to have the technical means to study the networks of two small structures essential for mounting and maintaining defensive responses to sustained threat. This study also informs the debate surrounding the role of CeA and BNST in fear vs. anxiety, while probing the extent to which the network contributions of these regions to the anxious state are overlapping or distinct. Results from a previous study demonstrated large subcortical network overlap between these two structures during rest^22^. Following the present anxiogenic manipulation, findings for both BNST and CeA point to a common alteration in connectivity with the vmPFC during the anxious state. Differences of IFCs between these regions are also noted. Threat-related decreases of IFC characterize BNST-cingulate cortex as well as BNST-nucleus accumbens couplings, while a threat-related increase is found with CeA–thalamus coupling. Finally, the examination of 109 ROI-IFCs (exploratory matrix) indicates that many other ROIs across the brain show increased couplings during threat. Of interest, the insula, a region prominently implicated in anxiety^48,74^ shows a threat-related increased IFC across the brain. Taken together, findings of this study support overlap and differences in connectivity between two anatomically related regions in response to threat, and suggest that multiple networks are simultaneously engaged during the anxious state.

## Electronic supplementary material

Supplemental material
