# Supplementary material for: Extended amygdala connectivity changes during sustained shock anticipation
Source: Transl Psychiatry. 2018 Jan 31;8:33. doi: 10.1038/s41398-017-0074-6 (PMC5802685; doi:10.1038/s41398-017-0074-6)
Supplement: Supplementary file 1 — Supplemental material [file 41398_2017_74_MOESM1_ESM.docx]

**Supplementary Methods and Figures for:***Extended amygdala connectivity changes during sustained shock anticipation* by Torrisi, et al.

**Exploratory correlation matrix construction**

The correlation matrix was assembled, using custom scripts, from a collection of regions of interest (ROIs) that were as subject-specific as possible, respectful of the high resolution data collected, and true to the acquired spatial coverage and temporal signal-to-noise they possessed. The procedure, which combined many regions and then pruned them accordingly, was as follows:

**Combining**

12 small regions of interest (dorsal nucleus of raphe, left and right anterior hippocampus, left and right posterior hippocampus, ventral and dorsal periaqueductal gray, lateral hypothalamus, habenula, centromedian thalamus and left and right dorsomedial thalamus) were chosen from our previous resting state study at ultra-high field (1). These regions exhibited strong positive functional connectivity with the BNST and were selected because no current atlases contain these regions and we wished to test how the functional ‘BNST network’, as well as how its coupling with cortical regions, changed under threat of shock. These masks were created with 2.8mm radius spheres around the published peak coordinates.

Additionally, the masks of 2 amygdala subnuclei (CeA region, bilaterally) were also added (2). The centers of mass of this amygdala subnucleus was visually checked against our template and an atlas in MNI space (3) and were found to be in agreement. Finally, 2 subject-specific, manually-drawn bilateral BNST masks were added to this collection of ROIs (1). Therefore, at this stage, each subject possessed a mask of 12+2+2=16 ROIs.

Next, subject-specific FreeSurfer (FS) parcellations and segmentations (4,5) were automatically processed from each individual’s T1-weighted structural scan. We chose the Destrieux FreeSurfer parcellation with greater density because (a) our data was high resolution, (b) we wished to retain anatomical interpretability, (c) available atlases, even when probabilistic, are based on other subjects and inevitably contain mismatches, (d) functionally-based parcellation techniques are still being worked out (6) and (e) it has been shown that higher density parcellations are more stable and robust in identifying brain states (7). These were added to the previous 16 ROIs.

**Pruning**

Subsequently, five steps of ‘ROI pruning’ were conducted. First, using the AFNI tool *3dmaskdump*, a list of each subject’s regions at this stage was calculated (208-211 ROIs). Those that FS had not identified in all 36 subjects were removed (values 30, 72) as well as 33 non-gray matter ROIs (values 2, 4, 5, 7, 10, 14, 15, 16, 17, 18, 24, 28, 31, 41, 43, 44, 46, 49, 53, 54, 60, 62, 63, 77, 80, 85, 251, 252, 253, 254, 255, 1000, 2000), leaving 174 cortical and subcortical regions. Note that to accommodate our coordinate-based spheres, i.e. the 12 functionally-defined regions listed above, the structural FS masks of these regions were also eliminated (i.e. FS’s two left and right “thalamus” masks were replaced by our four thalamic subnuclei listed above, the two left and right “amygdala” masks were replaced by the Tyszka masks, and the two “hippocampus” masks were replaced by our four bilateral anterior and posterior hippocampus masks). We note that non-FS ROIs were assigned integers values that had not already been allocated to FS regions^[[1]](#footnote-1)^. For the second pruning step, a binarized group mask was created at a threshold of 95% of a group average (Supplemental Figure 2). Our intent to acquire EPI data with both high spatial resolution and standard temporal resolution had necessitated a common trade-off of coverage, reflected in the thresholded group mask. This mask was then multiplied with the aforementioned 174 ROIs, which completely eliminated some ROIs (9 of them), cropped others and perfectly retained others still. 165 cropped and retained ROIs remained after this multiplication, as assessed with the tool *3dROIstats*. Because of the variable nature of the effects of the mask multiplication across subjects we then further eliminated up to 14 ROIs not shared by all.

Some ROIs still remaining, even though shared by all, however, were so severely cropped that to call them by their original anatomical labels was misleading and un-generalizable. Therefore, we were prompted to construct a third pruning criteria: the 160 ROIs remaining after the mask multiplication (and further trimming of inconsistency across subjects) were then measured as a percent of their pre-cropped masks, and if the group mean of these percents for any given ROI was less than 50% then that ROI was trimmed from all subjects. 121 ROIs remained following this step. Fourth, seven ROIs with very low average temporal signal to noise (TSNR; bottom 5^th^ percentile of all ROIs), due perhaps to size, location or iron deposition (8), were also eliminated, leaving 114. Fifth and finally, five ROIs contralateral to those which had been eliminated in one hemisphere were eliminated as well. This constraint to symmetry was to avoid lateralization in results due simply to EPI coverage. The remaining **109 ROIs** were used with AFNI’s FATCAT tools (9) to calculate matrices of functional connectivity differences between threat and safe conditions.

We believe that this procedure does the best with the data we possessed, given our data limitations, anatomical constraints and desire to include only regions of interest with good tSNR. In the near future we hope that standardized subcortical atlases will improve and be better suited to high-resolution data (eliminating the necessity of combining functionally with anatomically-defined masks), as well as coverage trade-offs will be minimized or eliminated with more advanced acquisition methods (10).

**Figures**


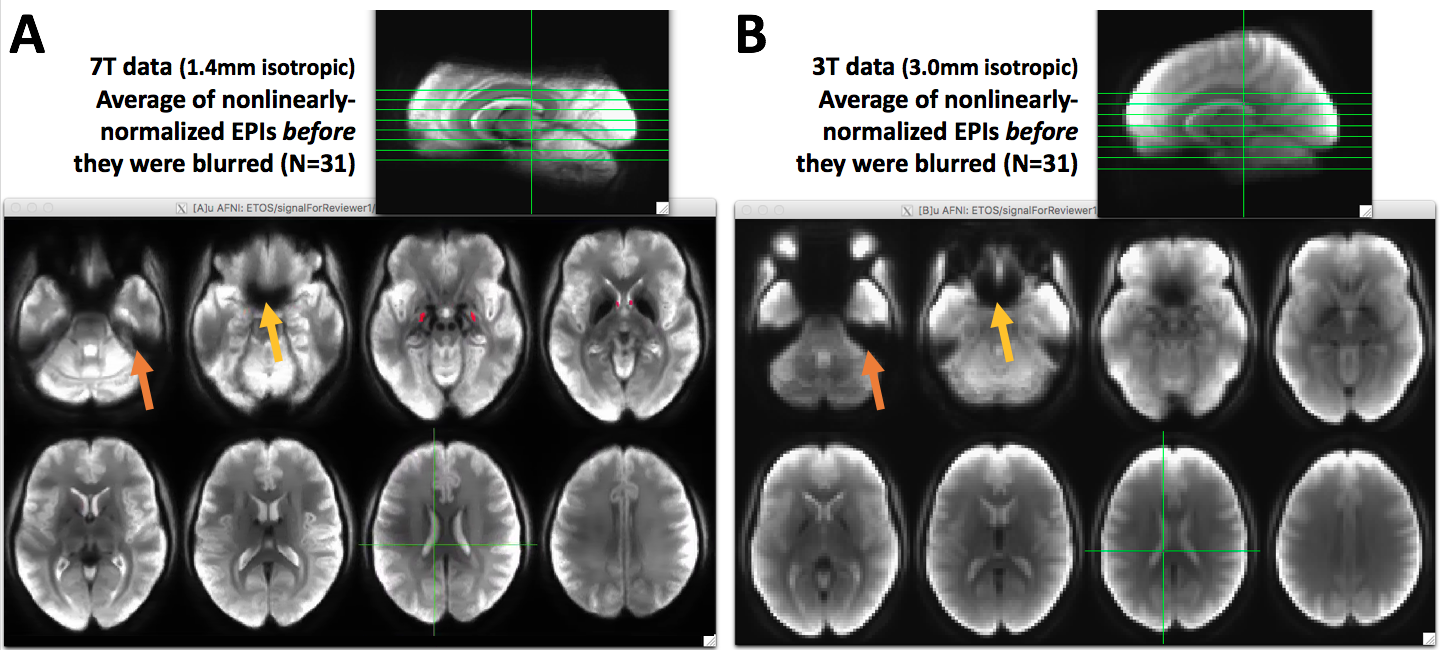
Supplemental Figure 1. Signal dropout between field strengths; acquiring thinner slices at 7T mitigates signal dropout (11,12). Panel A: mean of 31 of the 7T subjects’ nonlinearly-normalized *but unsmoothed* EPIs. BNST and CeA masks in red. Panel B: mean of a different 3T dataset (from (13)) with standard acquisition parameters. Both datasets were nonlinearly normalized with the same 3dQwarp algorithm to the same MNI space template. Orange arrows indicate dropout from ear canals, yellow arrows indicate dropout at orbitofrontal / vmPFC region.


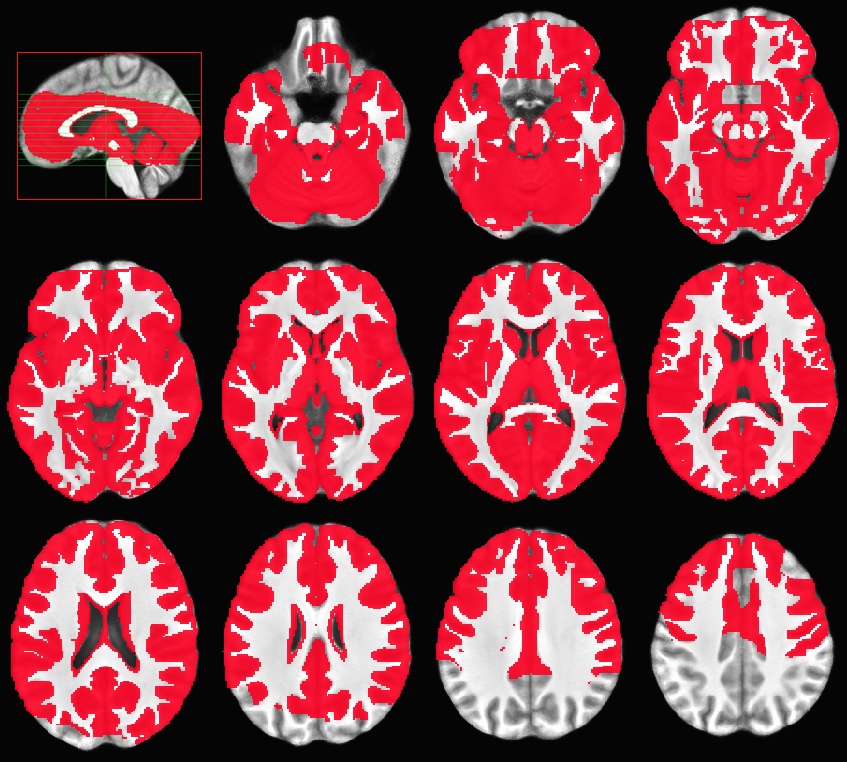


Supplemental Figure 2. The group mask that the voxel-level analyses were performed within. 25% probability gray matter mask from the template was additionally cropped to a field-of-view representing >=95% of subjects’ EPI coverage.

**REFERENCES**

1. Torrisi SJ, O'Connell K, Davis A, Reynolds R, Balderston N, Fudge JL, et al. Resting state connectivity of the bed nucleus of the stria terminalis at ultra-high field. Hum Brain Mapp. 2015 Jul 14;36(10):4076–88.

2. Tyszka JM, Pauli WM. In vivo delineation of subdivisions of the human amygdaloid complex in a high-resolution group template. Hum Brain Mapp. 2nd ed. 2016 Oct 6;37(11):3979–98.

3. Mai JK, Majtanik M, Paxinos G. Atlas of the Human Brain. Academic Press; 2015. 1 p.

4. Fischl B, Salat DH, Busa E, Albert M, Dieterich M, Haselgrove C, et al. Whole brain segmentation: automated labeling of neuroanatomical structures in the human brain. Neuron. Elsevier; 2002;33(3):341–55.

5. Destrieux C, Fischl B, Dale A, Halgren E. Automatic parcellation of human cortical gyri and sulci using standard anatomical nomenclature. Elsevier Inc; 2010 Oct 15;53(1):1–15.

6. Eickhoff SB, Thirion B, Varoquaux G, Bzdok D. Connectivity-based parcellation: Critique and implications. Hum Brain Mapp. 2015 Sep 27;36(12):4771–92.

7. Gonzalez-Castillo J, Hoy CW, Handwerker DA, Robinson ME, Buchanan LC, Saad ZS, et al. Tracking ongoing cognition in individuals using brief, whole-brain functional connectivity patterns. Proceedings of the National Academy of Sciences. 2015 Jun 29;:201501242.

8. Liem MK, Lesnik Oberstein SAJ, Versluis MJ, Maat-Schieman MLC, Haan J, Webb AG, et al. 7 T MRI reveals diffuse iron deposition in putamen and caudate nucleus in CADASIL. Journal of Neurology, Neurosurgery & Psychiatry. BMJ Publishing Group Ltd; 2012 Dec;83(12):1180–5.

9. Taylor PA, Saad ZS. FATCAT: (An Efficient) Functional And Tractographic Connectivity Analysis Toolbox. Brain Connectivity. 2013 Oct;3(5):523–35.

10. Moeller S, Yacoub E, Olman CA, Auerbach E, Strupp J, Harel N, et al. Multiband multislice GE-EPI at 7 tesla, with 16-fold acceleration using partial parallel imaging with application to high spatial and temporal whole-brain fMRI. Magn Reson Med. 2010 Apr 23;63(5):1144–53.

11. Iranpour J, Morrot G, Claise B, Jean B, Bonny J-M. Using High Spatial Resolution to Improve BOLD fMRI Detection at 3T. Yap P-T, editor. PLoS ONE. Public Library of Science; 2015 Nov 9;10(11):e0141358–15.

12. Olman CA, Davachi L, Inati SJ. Distortion and Signal Loss in Medial Temporal Lobe. García AV, editor. PLoS ONE. 2009 Dec 3;4(12):e8160–10.

13. Torrisi SJ, Robinson OJ, O'Connell K, Davis A, Balderston N, Ernst M, et al. The neural basis of improved cognitive performance by threat of shock. Social Cognitive and Affective Neuroscience. 2016 Jun 30;:nsw088–10.

1. see <https://surfer.nmr.mgh.harvard.edu/fswiki/FsTutorial/AnatomicalROI/FreeSurferColorLUT> [↑](#footnote-ref-1)
